# Hiatal Hernia Size and Reflux Parameters in Gastro-Oesophageal Reflux Disease: Evidence From a Retrospective Cohort

**DOI:** 10.7759/cureus.98131

**Published:** 2025-11-30

**Authors:** Mohammed Barghash, Emmanuel Obayi, Usifoh Itaman, Zoe Furber, Amy Caul, Ahmad Othman, Moustafa Mansour

**Affiliations:** 1 Upper Gastrointestinal Surgery, North Manchester General Hospital, Manchester, GBR

**Keywords:** demeester score, gord, hiatal hernia, pathological reflux, ph studies

## Abstract

Background

Hiatal hernia is commonly associated with gastro-oesophageal reflux disease (GORD). However, the relationship between hernia size and reflux severity remains unclear. This study aimed to assess the impact of the hiatal hernia size on DeMeester score, symptom correlation, and presence of pathological reflux on pH studies. The study also looked at the impact of various characteristics, including age, smoking, alcohol consumption, body mass index (BMI), and presence of oesophagitis and/or dysmotility, on DeMeester score, symptom correlation, and presence of pathological reflux on pH studies.

Methods

The study was conducted in an upper gastrointestinal surgery unit located in the North West of England. All patients aged 18 and above who underwent laparoscopic fundoplication for GORD between January 2017 and June 2023 were included.

Results

A total of 115 patients with a median age of 54 years were included. Patients' age was found to have a statistically significant association with hiatal hernia size above 2 cm, DeMeester score, and symptom association probability with p-values of 0.011, 0.021, and 0.030, respectively. Hiatal hernia size above 2 cm was found to have a statistically significant association with pathological reflux (p=0.017). The association of smoking with DeMeester score was found to be significant (p=0.027). Hiatal hernia size was not found to have a statistically significant association with DeMeester score, symptom index, or symptom association probability (p=0.115, p=0.315, and p=0.904, respectively).

Conclusion

Hiatal hernia size above 2 cm was found to have a statistically significant association with pathological reflux on pH studies, but there was no association found with elevated DeMeester score above 14.72 or symptom correlation indices.

## Introduction

Hiatal hernia is a prevalent condition characterised by the protrusion of the stomach through the diaphragmatic oesophageal hiatus into the thoracic cavity [1]. It is often associated with gastro-oesophageal reflux disease (GORD), which is a chronic disorder marked by the reflux of gastric content into the oesophagus [1]. The severity of GORD and its associated symptoms can vary widely among individuals. Therefore, a complete workup using 24-hour pH monitoring, oesophageal manometry, in addition to symptom evaluation, and endoscopy is mandatory to properly assess and diagnose patients with GORD [2].

Prolonged monitoring of oesophageal pH has demonstrated that brief episodes of reflux can be recorded in the majority of healthy subjects, especially in the post-prandial periods. Those episodes are considered physiological as long as they don't elicit symptoms or cause damage to the oesophageal mucosa. In contrast, pathological reflux is considered when reflux episodes are associated with symptoms or result in lesions in the exposed oesophageal mucosa [3].

In 1974, Johnson and DeMeester introduced a composite score that quantifies the extent of reflux over a 24-hour period via ambulatory pH monitoring [4]. Since then, the DeMeester score has been serving as a crucial parameter in evaluating the severity of GORD and guiding therapeutic decisions [4].

pH studies, in addition, have allowed the measurement of the association between the reported symptoms and the reflux events. Two of the most commonly used indices are the symptom index (SI) and the symptom association probability (SAP) [5,6]. For a long time, SI has been criticised because it does not take into account the total number of reflux events in its calculation. As such, SAP was introduced to serve as a better index for symptom correlation [7].

To our knowledge, the available evidence in literature on the association between hiatal hernia size and pathological reflux, DeMeester score, and symptom correlation indices is still limited. Therefore, in this study, we aimed to elucidate the impact of hiatal hernia size on DeMeester score, SI, SAP, and pathological reflux on pH studies. Additionally, we did seek to explore the influence of various demographic and clinical factors, such as age, smoking, alcohol consumption, body mass index (BMI), and the presence of oesophagitis and dysmotility, on DeMeester score, SI, SAP, and pathological reflux.

This article was previously presented as a meeting abstract at the 27th Association of Upper Gastrointestinal Surgery of Great Britain and Ireland (AUGIS) Annual Scientific Meeting on September 12, 2024 [8]. It was also published as a meeting abstract at the 20th World Congress for Esophageal Diseases hosted by the International Society for Diseases of the Esophagus (ISDE) on September 22, 2024 [9].

## Materials and methods

Study design and patient selection

We conducted a retrospective cohort study in compliance with the Strengthening the Reporting of Cohort Studies in Surgery (STROCSS) guideline for observational studies [10]. The study followed a predefined protocol that was compliant with the institutions' policies recommended by the local clinical governance unit. A research ethics committee approval was not required as the study had a retrospective design using non-identifiable hospital data. The study was conducted in the Upper Gastrointestinal Surgery of North Manchester General Hospital in Manchester, England. All patients aged 18 and above who underwent laparoscopic fundoplication for GORD between January 2017 and June 2023 were identified from the prospectively maintained electronic hospital database and were included. Patients who underwent fundoplication for para-oesophageal hiatal hernia, those who had fundoplication as a complementary step to Heller's cardio-myotomy, or those who underwent revisional surgery were excluded.

Comparisons and outcomes

The primary outcome was to compare patients diagnosed with a hiatal hernia size of more than 2 cm versus size of less than 2 cm in relation to the presence of pathological reflux on pH/impedance studies. The secondary outcome was to assess the associations between hernia size and DeMeester score [4], SI [5], SAP [6], and patient demographic/clinical characteristics (age, sex, BMI, smoking, alcohol, oesophagitis, dysmotility).

Hiatal hernia size and oesophagitis were assessed during upper gastrointestinal endoscopy. Hiatal hernia size was measured endoscopically using standard forward-view estimation of the distance between the diaphragmatic pinch and the squamo-columnar junction recorded in centimetres. DeMeester score, SI, SAP, and pathological reflux were concluded using validated published formulas following either 24-hour pH or impedance studies [4-6]. A DeMeester score of 14.72 and above was considered a positive surrogate to reflux. SI and SAP equal to or above 50% and 95%, respectively, were considered as positive symptom correlation [4-6]. Dysmotility was assessed and concluded using either high-resolution oesophageal manometry or barium swallow studies.

Data collection

An Excel sheet (Microsoft Corporation, Redmond, Washington, United States) was developed to collect the following data: patients' age in years, gender, smoking, alcohol consumption, BMI in kg/m², hiatal hernia size in centimetres, presence of oesophagitis, presence of dysmotility, DeMeester score [4], SI [5], SAP [6], and pathological reflux on pH studies. The data collection proforma used to extract variables is provided in the Appendices. DeMeester score [4], SI [5], and SAP [6] were calculated by the gastroenterology team using the standard published formulas. DeMeester score and SAP are available in open access [4,6]. The SI is a simple quantitative calculation (percentage of symptom-associated reflux events) and does not involve any copyrighted or proprietary material; therefore, permission for its use was not required [5].

Data synthesis and statistical analyses

The categorical variables were summarised using absolute and relative frequencies and were compared using the chi-squared test. The continuous variables were summarised using median (minimum-maximum) and were compared using the Mann-Whitney U test. All statistical tests were two-tailed, and statistical significance was assumed at p<0.05. The statistical analyses were performed using IBM SPSS Statistics for Windows, Version 25.0 (IBM Corp., Armonk, New York, United States).

## Results

Baseline patient characteristics

A total of 115 patients were included in the analysis. The cohort was middle-aged (median of 54 years) with a balanced gender distribution. The median BMI lay within the overweight range (27.95 kg/m²). The majority of patients had hiatal hernias larger than 2 cm (92.2%). On the other hand, oesophagitis (24.3%) and dysmotility (44.6%) were less common. Pathological reflux and elevated DeMeester scores were frequently observed (83.8% and 91.7%, respectively), whereas positive SI and SAP values were present in approximately more than half of the patients. Most patients ultimately underwent either Nissen (40.9%) or Toupet fundoplication (56.5%) (Table 1).

**Table 1:** Baseline characteristics of the included population N: number; SD: standard deviation; Min: minimum; Max: maximum; SI: symptom index; SAP: symptom association probability

Variable	N	Value (mean±SD or median (min-max))/n (%)
Age (years)	115	52.67±14.15 (median 54, range 22-80)
Gender: male	115	53 (46.1%)
Gender: female	115	62 (53.9%)
BMI (kg/m²)	112	28.19±3.76 (median 27.95, range 17.9-37)
Smoking: yes	112	38 (33.9%)
Smoking: no	112	74 (66.1%)
Alcohol: yes	112	66 (58.9%)
Alcohol: no	112	46 (41.1%)
Hiatal hernia size (cm)	115	median 3 (range 0-13); >2 cm: 106 (92.2%)
Oesophagitis: yes	115	28 (24.3%)
Dysmotility: yes	112	50 (44.6%)
Pathological reflux: yes	111	93 (83.8%)
DeMeester score ≥14.72	108	99 (91.7%)
SI ≥50%	93	58 (62.4%)
SAP ≥95%	94	54 (57.4%)
Operation: Dor	115	3 (2.6%)
Operation: Nissen	115	47 (40.9%)
Operation: Toupet	115	65 (56.5%)

Hiatal hernia size and its association with other different variables 

Hiatal hernia size greater than 2 cm showed a significant association with increasing age and with the presence of pathological reflux on pH monitoring (p=0.011 and p=0.017, respectively). No significant associations were found between hernia size and gender, BMI, smoking status, alcohol intake, oesophagitis, or dysmotility. Hernia size was also not associated with DeMeester score, SI, or SAP (Table 2).

**Table 2:** Association between hiatal hernia size and other different variables MWU: Mann-Whitney U test; X^2^: chi-squared test; BMI: body mass index; SI: symptom index; SAP: symptom association probability

Variable	P-value
Age (MWU)	0.011
Gender (X^2^)	0.424
Smoking (X^2^)	0.487
Alcohol (X^2^)	0.104
BMI (MWU)	0.144
Oesophagitis (X^2^)	0.335
Dysmotility (X^2^)	0.990
Pathological reflux (X^2^)	0.017
DeMeester score (X^2^)	0.115
SI (X^2^)	0.315
SAP (X^2^)	0.904

Pathological reflux, DeMeester score, and symptom correlation and their association with patients' basic characteristics 

Increasing age demonstrated significant associations with both elevated DeMeester scores and positive SAP results (p=0.021 and p=0.030, respectively). That suggests the possible age-related effect on reflux physiology. Smoking was also significantly associated with DeMeester score (p=0.027). No other demographic or clinical variables, including BMI, gender, alcohol intake, oesophagitis, or dysmotility, showed significant associations with pathological reflux, DeMeester score, SI, or SAP (Table 3).

**Table 3:** Association between patients' characteristics and pathological reflux, DeMeester score, SI, and SAP P: p-value; MWU: Mann-Whitney U test; X^2^: chi-squared test; BMI: body mass index; SI: symptom index; SAP: symptom association probability

Patient characteristic	Pathological reflux (P)	DeMeester score (P)	SI (P)	SAP (P)
Age (MWU)	0.091	0.021	0.281	0.030
Gender (X^2^)	0.313	0.907	0.400	0.514
Smoking (X^2^)	0.545	0.027	0.301	0.879
Alcohol (X^2^)	0.151	0.319	0.392	0.561
BMI (MWU)	0.353	0.914	0.688	0.241
Oesophagitis (X^2^)	0.560	1.000	0.936	0.965
Dysmotility (X^2^)	0.138	0.479	0.349	0.072

## Discussion

Our study highlighted a significant association between the size of the hiatal hernia and the presence of pathological reflux on pH studies. We found that hiatal hernias with a size greater than 2 cm were associated with pathological reflux (p=0.017). Although the link between hiatal hernia size and reflux is unclear in quantitative reflux studies, like multichannel intraluminal impedance-pH (MII-pH) studies, there is evidence in the literature demonstrating results similar to ours [11-13]. In a study by Franzén and Tibbling, they reported that patients with a large hiatal hernia were found to have much more acid reflux and reflux symptoms than those with small hernias [12]. In 2022, Shahsavari et al. associated a larger hiatal hernia size with a higher number of reflux episodes [13].

Despite the previous findings, we found no statistically significant association between hiatal hernia size and neither DeMeester score nor the symptom correlation indices such as SI and SAP. That is contrary to the findings published by Shahsavari et al., who found significant relationships between large hiatal hernias and higher DeMeester scores (p<0.01) [13]. While hiatal hernia size was not significantly associated with DeMeester score, the borderline p-value and the strong link with pathological reflux may suggest that the lack of significance may be due to the highly selected cohort or the rigid DeMeester score cut-off, rather than a true absence of association.

Additionally, our calculations did not prove any statistically significant association between hiatal hernia size and oesophagitis. Again, that is different from the already published data by Jones et al. in 2001, who reported that the size of the hiatal hernia correlated with oesophagitis severity (p=0.0001) [14]. Regarding oesophageal dysmotility, contrary to our findings, some studies suggested that hiatal hernia was linked to oesophageal dysmotility [4,15]. However, to our knowledge, there was no evidence to suggest any correlation between the size of the hernia and the degree of dysmotility.

One of the interesting theories that links hiatal hernia and GORD stated that hiatal hernia increases gastro-oesophageal reflux by widening the oesophageal hiatus, which in turn impairs the sphincteric function of the crural diaphragm. Subsequently, more oesophageal exposure to acid results in oesophagitis and subsequent fibrosis, which leads to oesophageal shortening. As such, the hiatal hernia increases in size, and the vicious cycle continues [1].

The association of hiatal hernia with age has also been previously investigated by many. Shahsavari et al., in their 2022 study, found that an increasing size of hiatal hernias was found in older patients [13]. Flora Filho and Zilberstein also stated in their cross-sectional study that age was significantly associated with the presence of hiatal hernias. Around 64.25% of the hernias in their study were in patients aged 54-64 years old [11]. In our study, age was significantly associated with hiatal hernia size above 2 cm, with a p-value of 0.011. Age was also found to be significantly associated with raised DeMeester score and SAP. The association between age and DeMeester score had also been recently proven by Shehata et al. and Pelzner et al., on Middle Eastern and German cohorts of patients, respectively [16,17]. Bearing that in mind, we can argue that age should be considered a strong risk factor for GORD. 

Many mechanisms of increasing incidence of gastro-oesophageal reflux in older age have been proposed. Impaired oesophageal motility, diaphragmatic weakness, and decreased lower oesophageal sphincter pressure in older age were argued to be contributing factors for reflux disease. Higher incidence of hiatal hernia and other comorbidities, including diabetes and Parkinson's disease, in older patients may also be considered [16].

Despite several studies looking at the hiatal hernia size and its impact on GORD, the novel feature in our study is looking at the impact of hiatal size on symptom correlation indices, including SI and SAP. On the other hand, our study is not without limitations. Those limitations include the retrospective nature of the study and all its inherited sources of bias. In addition, we used only the cohort of patients who underwent anti-reflux surgery, and that might be an underlying source of selection bias. Additionally, the hiatal hernia size was determined by endoscopy, which is operator-dependent. Finally, the study did not include blinding of data extractors. It also did not include an assessment of inter-observer reliability for endoscopic hernia measurements. This may introduce minor methodological bias.

## Conclusions

Hiatal hernias larger than 2 cm were significantly associated with pathological reflux but not with DeMeester score or symptom correlation indices. Increasing age was also associated with abnormal reflux parameters, suggesting a possible age-related effect on reflux physiology. Thorough standardised endoscopic and detailed physiological evaluation remains crucial when evaluating patients for anti-reflux surgery.

## Figures and Tables

**Figure 1:**
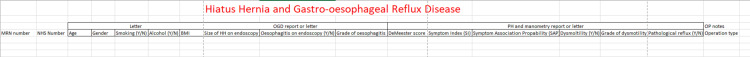
Data collection proforma (blank version used for recording study variables)
